# Patterns of Adaptive and Neutral Diversity Identify the Xiaoxiangling Mountains as a Refuge for the Giant Panda

**DOI:** 10.1371/journal.pone.0070229

**Published:** 2013-07-19

**Authors:** Yi-Yan Chen, Ying Zhu, Qiu-Hong Wan, Ji-Kang Lou, Wen-Jing Li, Yun-Fa Ge, Sheng-Guo Fang

**Affiliations:** The Key Laboratory of Conservation Biology for Endangered Wildlife of the Ministry of Education, State Conservation Center for Gene Resources of Endangered Wildlife, College of Life Sciences, Zhejiang University, Hangzhou, P. R. China; Ben-Gurion University of the Negev, Israel

## Abstract

Genetic variation plays a significant role in maintaining the evolutionary potential of a species. Comparing the patterns of adaptive and neutral diversity in extant populations is useful for understanding the local adaptations of a species. In this study, we determined the fine-scale genetic structure of 6 extant populations of the giant panda (*Ailuropoda melanoleuca*) using mtDNA and DNA fingerprints, and then overlaid adaptive variations in 6 functional *Aime*-MHC class II genes (DRA, DRB3, DQA1, DQA2, DQB1, and DQB2) on this framework. We found that: (1) analysis of the mtDNA and DNA fingerprint-based networks of the 6 populations identified the independent evolutionary histories of the 2 panda subspecies; (2) the basal (ancestral) branches of the fingerprint-based Sichuan-derived network all originated from the smallest Xiaoxiangling (XXL) population, suggesting the status of a glacial refuge in XXL; (3) the MHC variations among the tested populations showed that the XXL population exhibited extraordinary high levels of MHC diversity in allelic richness, which is consistent with the diversity characteristics of a glacial refuge; (4) the phylogenetic tree showed that the basal clades of giant panda DQB sequences were all occupied by XXL-specific sequences, providing evidence for the ancestor-resembling traits of XXL. Finally, we found that the giant panda had many more DQ alleles than DR alleles (33∶13), contrary to other mammals, and that the XXL refuge showed special characteristics in the DQB loci, with 7 DQB members of 9 XXL-unique alleles. Thus, this study identified XXL as a glacial refuge, specifically harboring the most number of primitive DQB alleles.

## Introduction

Genetic variability plays an important role in maintaining the evolutionary potential of a species. Neutral molecular markers can be used to determine neutral genetic diversity patterns, and deduce genetic structure and population history. As various selective forces acting on functional genes in natural populations could diversify adaptive variability [1]–[3], adaptive markers with fitness consequences other than neutral markers should be used to reveal the patterns of adaptive variation. To better understand the evolutionary potential of a species and the local adaptation features of populations, it is necessary to evaluate the diversity patterns and the association between the neutral and adaptive variations within extant populations.

**Figure 1:**
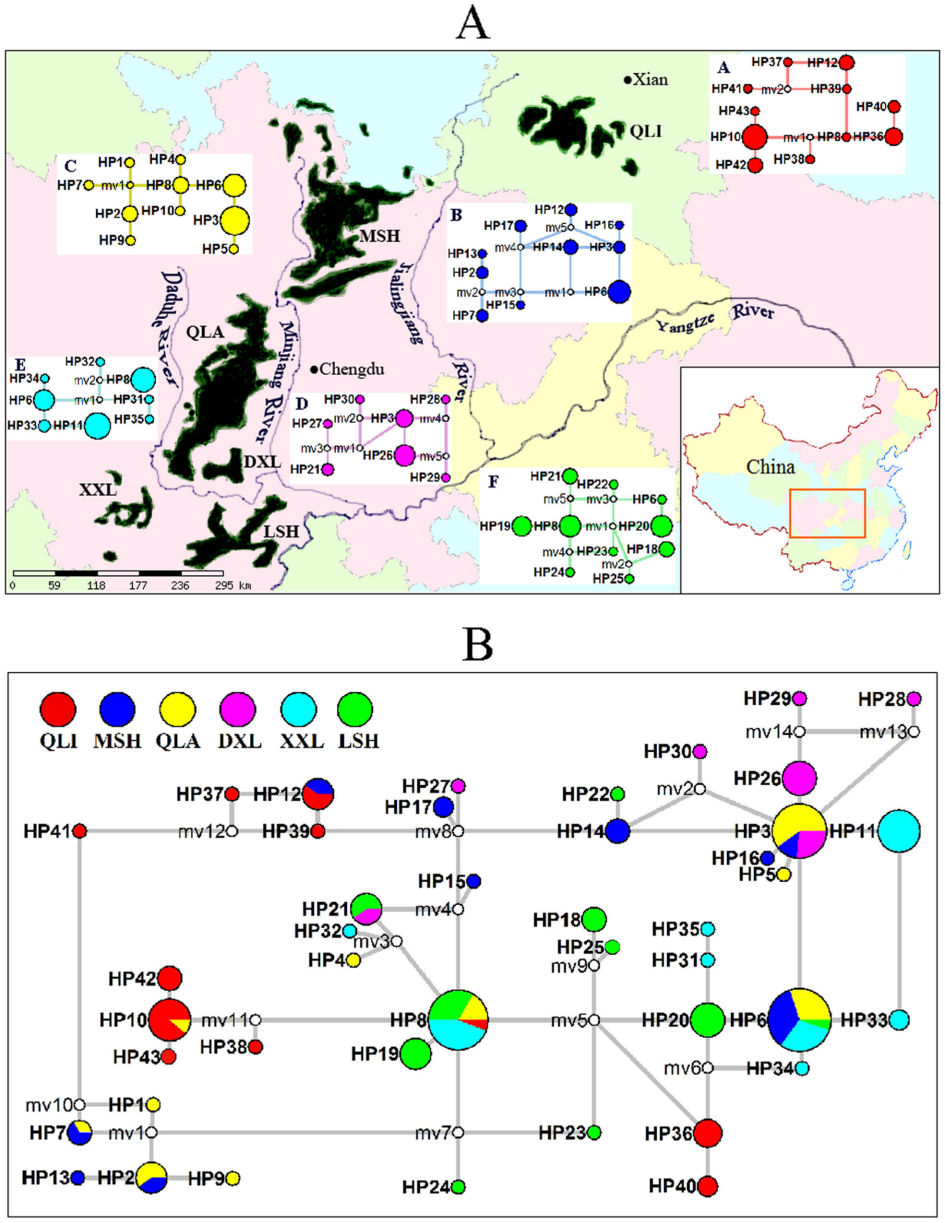
Distribution of giant panda populations and network relationships among panda mtDNA haplotypes. Current distribution of extant giant panda populations (A) and network relationships among the panda mtDNA haplotypes (B). The 6 isolated populations are indicated in dark green, according to the most recent survey [5]. Population-scale networks are shown in a and b (QLI, red; MSH, blue; QLA, yellow; DXL, purple; XXL, sky-blue; and LSH, green). The solid circles represent each unique haplotype, with their sizes proportional to their frequency. Empty circles indicate the undetected haplotypes that are necessary to link all observed haplotypes to the network.

The giant panda (*Ailuropoda melanoleuca*, Ursidae, Carnivora) is an ancient species once widely distributed throughout eastern and southern China, extending to northern Burma and northern Vietnam [4]. However, due to habitat loss from increasing and human continuing activities, the species is currently isolated as 6 extant populations in the Qinling (QLI), Minshan (MSH), Qionglai (QLA), Daxiangling (DXL), Xiaoxiangling (XXL), and Liangshan (LSH) mountain ranges on the edge of the Tibetan plateau in China [4] (Figure 1A). The sizes of these giant panda populations range from 29 to 708, with ∼1,600 total individuals [5], making it one of the world's most endangered species.

Previous studies based on neutral markers showed that low to moderate levels of genetic diversity [6]–[13] were preserved in the giant panda with limited gene flow [14], [15]. While significant divergence was detected among populations [9], [13], a new subspecies (the Qinling subspecies, *Ailuropoda m. qinlingensis*) was recognized from the nominate subspecies (*Ailuropoda m. melanoleuca*) [16], [17]. The abovementioned previous studies focused on genetic variation within certain populations rather than in all 6 populations, except the DNA fingerprinting study of Wan *et al.* [16]. Therefore, the first objective of this study was to define the fine-scale genetic structure of the 6 extant populations using mtDNA (control region) and DNA fingerprinting markers. The fingerprinting data of Wan *et al.* [16] were also reanalyzed to address the population history of the giant panda.

The major histocompatibility complex (MHC) genes play an essential role in the adaptive immune system of vertebrates [18]. The antigen binding regions of MHC molecules, which are involved in pathogen recognition, are highly polymorphic [1], [18]–[20]. Even species that are genetically monomorphic at neutral markers have a high level of polymorphism at MHC loci [21]. Therefore, MHC has become a functionally important marker system in the analysis of adaptive variation in animals [1], [18], [19]. The 3 major hypotheses [22] that have been suggested for the maintenance of the adaptive polymorphism of the MHC are overdominance [23], rare-allele advantage [24], and spatio-temporal selection [25].

Many studies of *Aime-*MHC class II genes [26]–[30] have been performed, and, by the HURRAH method, a total of 6 classical MHC class II loci have been confirmed to be expressed (see the supporting information section in Wan *et al.* [31]); these loci are linked on chromosome 9q in the following order DRA-DRB3-DQA1-DQB1-DQA2-DQB2 [26], [28], [29], [31]. These detailed genomic data lead to the development of a suite of methods for polymorphism (SSCP) and sequence analysis [30]. Therefore, based on the well-developed genotyping techniques of these *Aime*-MHC genes, the second objective of this study was to understand how the patterns of MHC-based adaptive variation vary in relation to the patterns of neutral genetic variation within wild populations, and what adaptation strategies are adopted by the giant panda compared to other carnivores.

## Materials and Methods

### Ethics statement

The samples used in the present study were all collected from wild individuals, including blood, liver, skin, and feces. All blood samples were obtained from wild-born captive giant pandas during their routine medical examinations, and the 3 liver samples were obtained from dead wild (rescued) pandas that died from ascariasis. These wild-born pandas were housed in the China Research and Conservation Center for the Giant Panda (Wolong) for routine examination or before their death. Wolong collected these samples as gene resources with permission from the China Giant Panda Protection and Management Office (CGPPMO) and deposited them in the State Conservation Center for Gene Resources of Endangered Wildlife of China (SCCGREWC).

Skin tissues were collected from dead wild pandas at different nature reserves (NRs) over the past decades, including Foping NR, Zhouzhi National NR, Baishuijing National NR, Wanglang NR, Tangjiahe NR, Wolong NR, Baoxing NR, Yele NR, Dafengding NR, Heizhugou NR, and the Louguantai Wild Animal Breeding and Protection Center. The causes of death were mostly natural; a few deaths were due to infectious diseases such as ascariasis, pneumonia, and tick-borne diseases. These NRs obtained permission from CGPPMO to collect these samples as genetic resources and delivered them to SCCGREWC for preservation.

Feces samples were collected from the Louguantai Wild Animal Breeding and Protection Center (QLI region), the China Research and Conservation Center for the Giant panda (MSH region), Daxiangling NR (DXL region), Yele NR (XXL region), Liziping NR (XXL region), Dafengding NR (LSH region), and Heizhugou NR (LSH region). We obtained specific permission from the Louguantai Wild Animal Breeding and Protection Center and China Research and Conservation Center for the Giant Panda to take fecal samples from wild-captured captive individuals. We were authorized by the CGPPMO to collect the fecal samples from the DXL, XXL, and LSH regions during the non-reproductive season. We confirmed that we did not impact the animals during sampling. These feces were also stored at the SCCGREWC. We obtained permission from the SCCGREWC to use the above-mentioned samples in this study.

### Sampling and DNA extraction

In total, 292 wild-born panda samples from 243 individuals were collected from 6 natural populations in different mountain ranges (see Table S1 in Supporting information; Figure 1A). The sample types included blood, feces, liver, and skin (Table S1 in Supporting Information). For the fecal samples, the outer layers were peeled from fresh feces and stored in 95% ethanol. Fecal samples of the QLI population were obtained from wild-captured pandas of known identity housed in the Louguantai Wild Animal Breeding and Protection Center, while MSH fecal samples were collected from animals of known identity housed at the China Research and Conservation Center for the Giant Panda. The remaining 123 fecal samples, which were from 74 individuals from DXL, XXL, and LSH and were confirmed based on MHC genotyping and sampling information, were field samples from the various mountain ranges obtained between August and November in 2009.

Genomic DNA was extracted from blood, liver, and skin samples using the standard phenol/chloroform method [32]. To extract DNA from the fecal samples, the ethanol was first dried under a vacuum, and then genomic DNA was isolated using the EZNA Stool DNA Kit (Omega Bio-tek, Inc., Norcross, GA) along with negative controls according to the manufacturer's instructions.

### Amplification and sequencing of mtDNA

A 706∼708-bp fragment of the hypervariable 5′ end of the mtDNA control region was amplified using primers (Table S2 in Supporting information) designed based on the mtDNA genome of the giant panda (GenBank accession no. EF196663). We used routine PCR and bi-directional sequencing. Ultimately, good sequencing data from 149 individuals were analyzed.

### Amplification and genotyping of MHC class II loci

Locus-specific primer sets were used to amplify exon2 of 6 functional *Aime-*MHC class II loci: DRA, DRB3, DQA1, DQA2, DQB1, and DQB2 (Table S2 in Supporting information). PCR amplification were performed according to the method of Chen et al. [30], GC buffer was used for DQB1 and DQB2 due to the high GC content of the target fragments. For the fecal samples, a multiple-tube procedure [33] was used to obtain reliable genotypes. SSCP genotyping and identification of MHC alleles were performed according to the method of Chen *et al.* [30]. For each MHC locus, an average of 18 individuals from each population were sequenced, and a total of 1,420 clones were analyzed. Sequences were validated as genuine alleles according to the criteria summarized by Kennedy *et al.* [34]. And in our study, the term “allele” was used for unique sequence variants.

### Data analysis

#### mtDNA, DNA fingerprinting, and population structure

MtDNA sequences (GenBank accession nos. JQ975131–JQ975173) were edited and aligned using the MEGA5 program [35]. Haplotypes were defined and their population frequencies were determined. To investigate the phylogenetic relationships of the mtDNA haplotypes, the sequences were used to construct network trees with the median-joining method [36] using NETWORK 4.5.1.6 (Fluxus Technology Ltd., Suffolk, UK). Genetic differentiation was assessed with Jost's D (*D*_est_) [37], and was calculated using the online program SMOGD version 1.2.5 [38] with 1,000 bootstraps. Mantel tests performed with the Isolde program, implemented in Genepop version 4.0.10 [39], were used to test for significant correlations between geographical and genetic distances with 100,000 permutations. Possible historic population size changes were detected by examining the pairwise mismatch distributions [40] of the panda mtDNA haplotypes, as calculated in DNASP 4.50.3 [41]. In addition to mismatch analysis, we performed Bayesian skyline plot analysis to estimate the dynamics of population size fluctuation over time with 10,000,000 Markov Chain Monte Carlo (MCMC) generations using BEAST v. 1.7.5 [42]. The HKY model was chosen as suggested by Jmodeltest 1.4.0 [43]. We used the relaxed molecular clock models with a rate of 30% substitutions per nucleotide site per million years for the control region [44]. Convergence of the chains was inspected using Tracer v. 1.5 [45].

As mtDNA is maternally inherited as a single locus, even the non-coding regions (such as control regions) might be subjected to background selection, and it might not completely reflect a species' population history, especially the demographic events caused by biparental inheritance. Therefore, it is necessary to examine nuclear markers in parallel with mtDNA sequences. Here, we reanalyzed DNA fingerprinting data from 49 individuals previously published by our colleagues [16] to assess the population history of extant giant pandas. The multilocus DNA fingerprints were produced by hybridization of a oligonucleotide probe (gp2000: (CTCCACCT)_3_) [46] with digested genomic DNA from wild pandas [16]. The Data was used to reconstruct a median-joining network tree using NETWORK 4.5.1.6 and maximum parsimony (MP) trees with 2,000 bootstrap replicates using PAUP* 4.0b10 [47].

#### Adaptive diversity of Aime-MHC class II genes

The obtained sequences were edited and aligned using MEGA5 software. The amino acid sequences of *Aime-*MHC alpha and beta alleles were then aligned with their HLA equivalents as references (GenBank accession nos.: HLA-DQA1, DQ284439; HLA-DRA, NM_019111; HLA-DQB1, AM259941; HLA-DRB3, and NM_022555). Pairwise and overall differences among the nucleotide and amino acid sequences were calculated for each locus and across genes. Antigen binding sites (ABS) were predicted based on comparison to homologous HLA molecules [20]. We checked for evidence of positive selection using 2 methods. First, we calculated the ratios of non-synonymous (*d*_N_) and synonymous (*d*_S_) substitutions for ABS sites, non-ABS sites, and all sites, with standard errors computed using 1,000 bootstrap replicates. The significances of the differences between *d*_N_ and *d*_S_ were estimated by Z-test analyses of selection using the modified (R = 2.3) Nei-Gojobori model [48]. Second, we used the maximum-likelihood (ML) method in the CODEML program of PAML4.1 [49]. The models considered in this study were M1a (nearly neutral), M2a (positive selection), M7 (nearly neutral with beta), and M8 (positive selection with beta and ω) [50]. The models M1a vs. M2a and M7 vs. M8 were usually compared in pairs to test for positive selection. In the present study, models M7 vs. M8 is powerful to detect positive selection than M1a vs. M2a; therefore, we only displayed the results of M7 vs. M8. We used the likelihood-ratio test (LRT) [51] to compare these 2 models to infer positive selection. In addition, we conducted Bayes empirical Bayes Bayesian (BEB) analysis to identify codons under positive selection in model M8 [52].

To analyze the allelic relationships among giant panda MHC genes, maximum likelihood (ML) phylogenetic trees were constructed for the alpha and beta genes using PhyML 3.0 [53] with 1,000 bootstrap replicates using the best-fit nucleotide substitution models as evaluated in Jmodeltest 1.4.0 [43]. The GenBank accession numbers for 46 panda alleles were GQ496164–GQ496188 and JN255198–JN255218. The following sequences from related mammals were downloaded as references: *Ursus arctos*: Urar-DQA*01–03 (AB378100-2), Urar-DQA*05 and 06 (JX469890-1), Urar-DQB*01–04 (JX469892-5), Urar-DRB*11, Urar-DRB*13 [54]; *Canis familiaris*: DLA-DQA (AF343734), DLA-DRA (L37332), DLA-DQB1 (DQ528655), DLA-DRB (AY220509); *Zalophus californianus*: Zaca-DQA (AF502560), Zaca-DRA (AY491453), Zaca-DQB1*01 (AF503397), Zaca-DRB (AY491465); *Homo sapiens*: HLA-DQA1 (NM_002122); HLA-DRA (NM_019111); HLA-DRB1 (NM_002124) and HLA-DRB5 (AK314834); *Felis catus*: FLA-DRA (EU915362); *Ursus thibetanus*: Urth-DQB (AB473936); *Urocyon littoralis*: Urli-DQB (AY366484); *Mustela lutreola*: Mulu-DRB (EU263556); *Ursus maritimus*: Urma-DRB (AF458937).

The distributions of the allele frequencies for the MHC genes were calculated using Fstat 2.9.3 [55]. Observed heterozygosity (*H_O_*) and deviation from Hardy-Weinberg Equilibrium were estimated using a Markov chain calculated in Arlequin ver 3.5.1.2 [56]. Population differentiation estimator *D*_est_ [37] was calculated across MHC class II loci using the online program SMOGD ver. 1.2.5 [38] with 1,000 bootstraps. Mantel tests were also conducted for MHC genes. Both the arithmetic and harmonic means of *D*_est_ across MHC loci were used to assess the Mantel tests.

## Results

### MtDNA haplotypes of the giant panda

**Figure 2:**
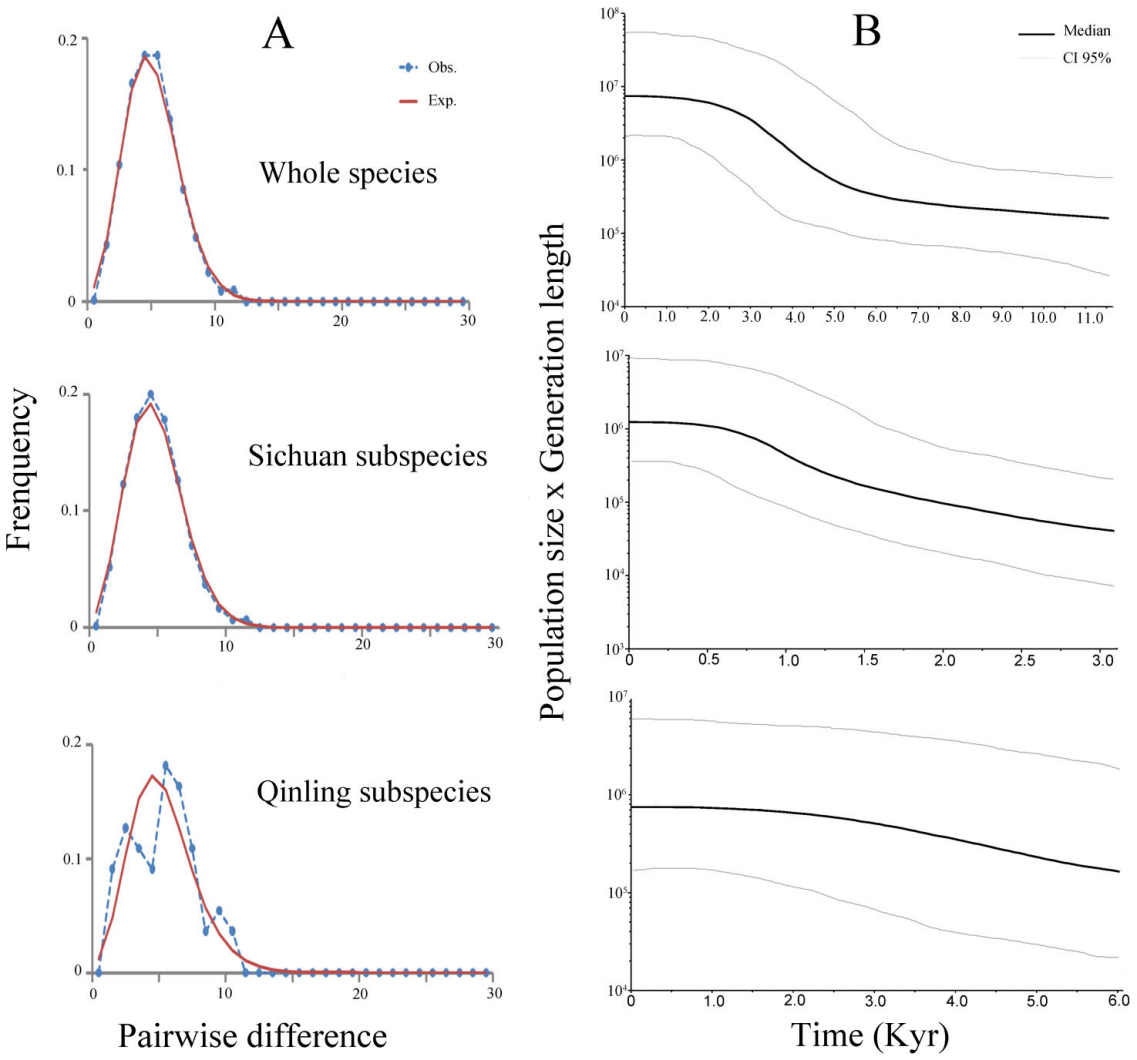
MtDNA-based mismatch distributions and Bayesian skyline plot. MtDNA-based mismatch distributions (A) and Bayesian Skyline Plot (B) for the 2 subspecies and the species as a whole.

We detected a total of 43 mtDNA haplotypes in the 6 panda populations, with 7 to 11 haplotypes in each population (Figure 1A, Table S3 in Supporting Information). Relatively frequent haplotypes were found in each population (Figure 1A), and 8 haplotypes were shared among the populations, which was reflected in the median-joining network generated using these data (Figure 1B). The remaining 35 haplotypes were population-specific (QLI, 8; MSH, 5; QLA, 4; LSH, 7; DXL, 5; XXL, 6; Figure 1B, Table S3 in Supporting Information). Among the 8 shared haplotypes, HP6 and HP8 were widely distributed with high frequencies in 4 populations while the other 6 haplotypes were shared by at least 2 populations (Figure 1B). Collectively, the mtDNA-based network depicts possible gene flow among the populations, and 3 unique, haplotype-rich populations, QLI, XXL, and LSH (Figure 1B). From the mismatch distribution of mtDNA haplotypes, we observed smooth, unimodal (bell-shaped) distributions of the species and the Sichuan subspecies, and a ragged profile for the Qinling subspecies (Figure 2A), which reflect the characteristics of expanding (for the Sichuan subspecies) and stable (for the Qinling subspecies) populations [40], respectively. In addition, we observed unimodal curves for the XXL population (data not shown), suggesting expansion of XXL and its potential special status in the Sichuan subspecies. The Bayesian skyline plot analysis of mtDNA showed that the whole species had a population expansion approximately 5,000 years ago (Figure 2B). When the 2 subspecies were analyzed separately, expansion was clearly observed in the Sichuan subspecies, whereas a relatively constant population size was observed between 1,000 to 3,000 years ago in the Qinling subspecies (Figure 2B). These results were in accordance with those based on mismatch distribution of mtDNA, but not exactly in accordance with recently published whole-genome resequencing results [57]. The resequencing results indicated that the MIN and QLA-DXL-XXL-LSH populations increased (referred to as the Sichuan subspecies in the present study) whereas the QIN population (the Qinling subspecies) decreased within this time period. The difference between the 2 results in the QIN population might be attributed to differential sensitivity of these two suites of markers. The Bayesian skyline plot in this study was based on maternally-inherited mtDNA, whereas the demographic history curve in the resequencing study was derived from genome-wide biparental single nucleotide polymorphisms [57].

### Population history analysis using previously reported DNA fingerprinting data

**Figure 3:**
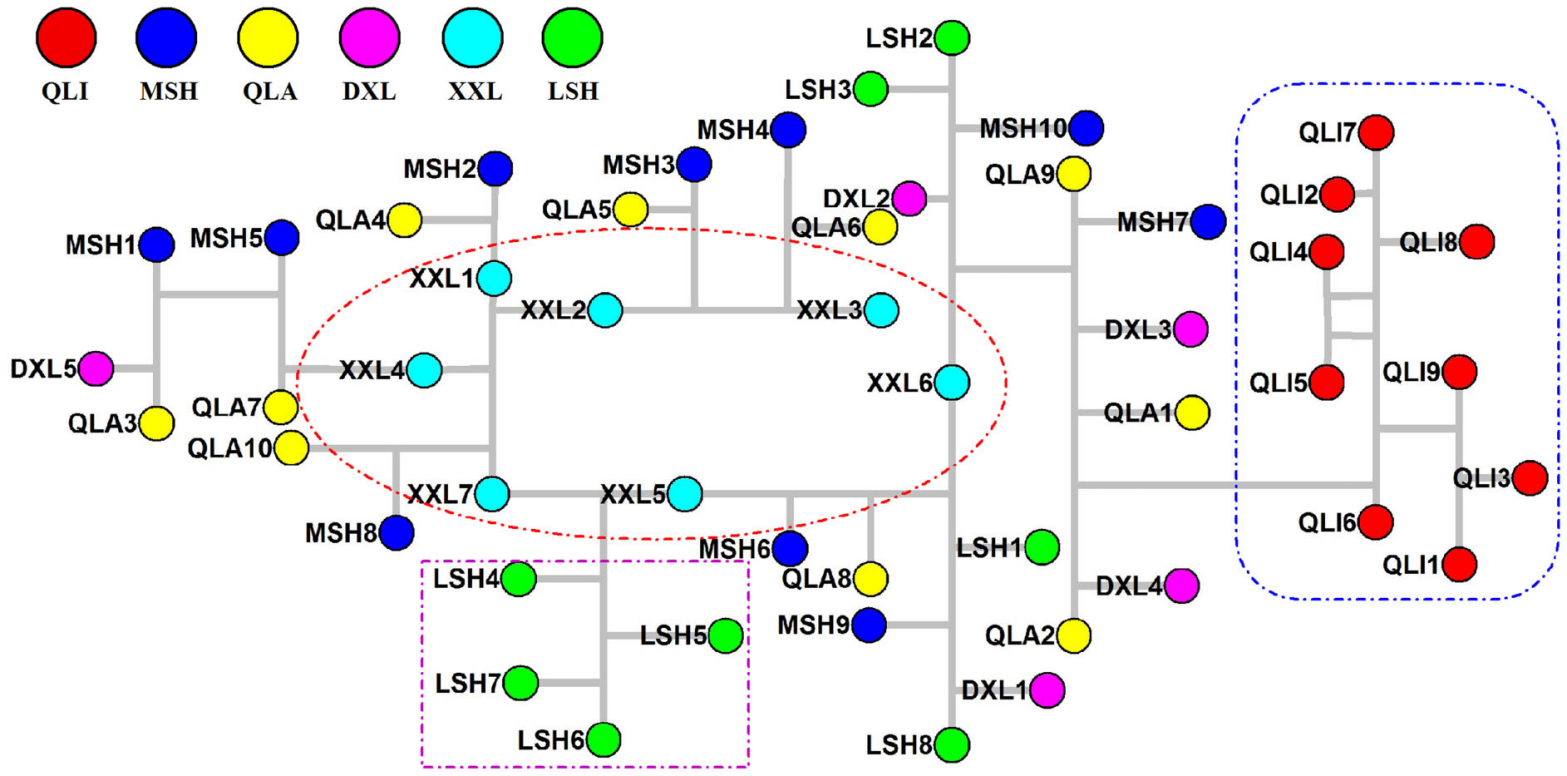
DNA fingerprint-based median-joining network. DNA fingerprint-based median-joining network relationships of the 6 panda populations. The populations from which the individuals were collected are indicated in the same color scheme given in Figure 1B).

In the median-joining network tree constructed from multilocus DNA fingerprints, all individuals from the 5 populations of the Sichuan subspecies formed a major clade, while the 9 individuals from the Qinling subspecies comprised a unique and distinct clade (Figure 3). Within the Sichuan subspecies clade, all individuals from the XXL population were located in the center. In addition, the fingerprinting tree revealed that half of the individuals from the LSH population (LSH4, LSH5, LSH6, and LSH7; Figure 3) formed a distinct cluster between individuals XXL5 and XXL7, suggesting the partially independent evolutionary history of the LSH population. Nonetheless, most of the bootstrap values were lower than 50% for the MP trees; therefore, we did not show the data here.

### Sequence variations of the Aime-MHC class II loci

For the 6 functional *Aime-*MHC class II loci, a total of 46 alleles were identified in the 6 extant panda population, with 2 to 12 alleles per locus (Table S4 in Supporting Information). Nineteen alleles were newly isolated in the present study (Tables 1 and 2, Figure S1A and S1B in Supporting Information), whereas the other alleles were identical to those reported in previous studies [30], [31]. We detected no more than 2 alleles in any individual at any locus, and we did not find any stop codons, insertions, deletions, or frame-shift mutations in any allele. DRA and DQB2, which were reportedly monomorphic in captive populations [30], were found to be dimorphic and trimorphic, respectively, in wild populations. The alpha genes (DRA, DQA1, and DQA2) were moderately divergent, whereas the beta genes (DRB3 and DQB1) were highly divergent (Table S4, Figure S1A and S1B in Supporting Information). Despite the remarkable sequence divergence detected across the loci, 7 pairs of allele sequences differed by only 1 nucleotide (Table S4, Figure S1A and S1B in Supporting Information).

**Table 1:** Allele frequencies, numbers of alleles and observed heterozygosities (*H_O_*) for the *Aime*-MHC class II alpha genes.

Locus	Allele a	Population						Locus	Allele a	Population					
		QLI	MSH	QLA	DXL	XXL	LSH			QLI	MSH	QLA	DXL	XXL	LSH
DQA1	DQA1*01	0.04	0.02	0.32	0.22	0.34	0.30	DQA2	DQA2*01	0.48	0.75	0.83	0.28	0.50	0.53
	DQA1*02	0.18	0.45	0.13	0.13	0.05	0.07		DQA2*02	0.02	0.19	0.16	0.44	0.38	0.38
	DQA1*03	0.31	0.02	0.18	0.09	0.08	0.13		DQA2*03	0.23	0.06	0.02	0.06	0.07	0.01
	DQA1*04	0.07	0.06	0.09	0.09	0.16	0.21		DQA2*04 c	0.28	–	–	–	–	–
	DQA1*05	0.28	0.23	0.25	0.25	–	0.09		**DQA2** * **05**	–	–	–	0.03	–	0.07
	DQA1*06	0.06	0.04	0.02	0.09	0.13	–		**DQA2** * **06**	–	–	–	0.09	–	0.01
	DQA1*07	0.06	0.05	0.02	–	–	0.05		**DQA2** * **07**	–	–	–	0.06	0.03	–
	**DQA1** * **08**	0.01	–	–	0.03	–	0.04		**DQA2** * **08**	–	–	–	0.03	0.02	–
	**DQA1** * **09**	–	0.12	–	0.09	0.25	0.07		*H_O_*	0.28**	0.32	0.33	0.81	0.74	0.71
	**DQA1** * **10** b	–	–	–	–	–	0.05								
	*H_O_*	0.85*	0.68*	0.79	0.80	0.81**	0.90								
DRA	DRA*01	0.98	0.99	1.00	1.00	0.85	0.92	all	DQA (18)	12	11	10	15	11	14
	**DRA** * **02**	0.02	0.01	–	–	0.15	0.08		DRA (2)	2	2	1	1	2	2
	*H_O_*	0.00*	0.03	–	–	0.10	0.10		All (20)	14	13	11	16	13	16

Numbers in parentheses are the total numbers of alleles.

*: *P*<0.05 and **: *P*<0.01.

a Alleles represented in bold were newly identified in current study, while the others were defined in a previous study [30];

b and ^c^ indicate the allele was LSH-specific and QLI-specific (Qinling subspecies-specific), respectively.

**Table 2:** Allele frequencies, numbers of alleles and observed heterozygosities (*H_O_*) for the *Aime*-MHC class II beta genes.

Locus	Allele a	Population						Locus	Allele a	Population					
		QLI	MSH	QLA	DXL	XXL	LSH			QLI	MSH	QLA	DXL	XXL	LSH
DQB1	DQB1*01	0.01	–	0.09	–	0.09	0.11	DRB3	DRB3*01	0.35	0.13	0.22	0.20	0.23	0.20
	DQB1*02	0.03	0.04	0.31	0.54	0.18	0.32		DRB3*02	0.03	–	0.03	–	0.02	0.03
	DQB1*03	–	0.07	0.09	–	0.12	0.09		DRB3*03	0.04	0.17	0.09	0.15	0.22	0.34
	DQB1*04	0.76	0.53	0.38	–	0.29	0.41		DRB3*04	0.33	0.16	0.06	–	–	0.06
	DQB1*05	0.20	0.26	0.09	–	–	0.012		DRB3*05	0.06	0.11	0.09	0.10	0.07	0.04
	DQB1*06	–	0.06	0.02	–	0.03	–		DRB3*06	0.04	–	–	0.30	–	–
	**DQB1** * **07** b	–	0.04	0.03	0.46	0.06	0.05		DRB3*07	0.06	0.20	0.02	–	–	0.03
	**DQB1** * **08** c	–	–	–	–	0.03	–		DRB3*08	0.02	0.07	0.22	–	0.12	0.03
	**DQB1** * **09** c	–	–	–	–	0.03	–		**DRB3** * **09**	0.07	0.17	0.28	0.25	0.23	0.27
	**DQB1** * **10** c	–	–	–	–	0.12	–		**DRB3** * **10** c	–	–	–	–	0.05	–
	**DQB1** * **11** c	–	–	–	–	0.03	–		**DRB3** * **11** c	–	–	–	–	0.07	–
	**DQB1** * **12** c	–	–	–	–	0.03	–		*H_O_*	0.83	0.82	0.74	0.30**	0.73	0.89**
	*H_O_*	0.43	0.33**	0.56*	0.64	0.77*	0.75**								
DQB2	DQB2*01	1.00	1.00	1.00	1.00	0.91	1.00	all	DQB (15)	5	7	8	3	14	7
	**DQB2** * **02** c	–	–	–	–	0.05	–		DRB (11)	9	7	8	5	8	8
	**DQB2** * **03** c	–	–	–	–	0.05	–		all (26)	14	14	16	8	22	15
	*H_O_*	–	–	–	–	0.09*	–								

*: *P*<0.05.

**: *P*<0.01.

Numbers in parentheses are the total numbers of alleles.

a The alleles bolded were newly identified in the current study, while the others were defined in previous studies [30], [31];

b and ^c^ indicate the allele was Sichuan subspecies-specific and XXL-specific (refuge), respectively.

### Population variation in the Aime-MHC class II loci

Most of the *Aime-*MHC alleles identified in the present study were shared among the 6 populations. However, there were 11 alleles that were unique to particular populations (DQA1*10 in LSH, DQA2*04 in QLI, and 9 in XXL (Tables 1 and 2). Hence, 24 to 35 MHC alleles were detected in each of the 6 panda populations (QLI, 28; MSH, 27; QLA, 27; DXL, 24; XXL, 35; and LSH, 31). More DQ alleles than DR alleles were identified in all populations (Tables 1 and 2).

The allele frequency of *Aime*-MHC class II loci is shown in Tables 1 and 2. There was a major population-level difference in allele frequency at DRB3 between the Qinling and Sichuan subspecies. Among the 18 DQA alleles, DQA2*01 was predominant in all panda populations except in DXL. The allele frequency distribution of the DQB genes showed the following (Table 2): (1) the DQB alleles were widely distributed in XXL (which contained 14 of the 15 alleles for this locus, including 7 unique alleles), revealing an MHC diversity center for the giant panda; (2) the DQB1*07 allele was unique to the Sichuan subspecies; (3) only 2 DQB1 alleles (DQB1*02 and DQB1*07) were present in DXL, possibly reflecting allele loss due to a bottleneck or demographic fragmentation in this small population. Finally, DRA*01 was frequent in all populations (0.85–1.0), whereas DRA*02 was present at low frequencies in QLI, MSH, XXL, and LSH (Table 1).

Heterozygosity diverged greatly across the different *Aime-*MHC loci and panda populations (Tables 1 and 2). High heterozygosity was consistently detected at DQA1 (0.68–0.90; Table 1) and DRB3 (0.73–0.89, Table 2). Lower heterozygosities of DQA2 and DQB1 were observed in the 3 larger populations (QLI, MSH, and QLA; ≤0.56), whereas higher heterozygosities were observed in the 3 smaller populations (DXL, XXL, and LSH; ≥0.64; Tables 1 and 2). The heterozygosities for DRA and DQB2 were very low, if present at all (0.0–0.1; Tables 1 and 2). Two of the smaller populations, XXL and LSH, displayed exceptionally high diversities (≥0.70) compared to the other populations at 4 loci.

### Genetic differentiation revealed by Aime-MHC and mtDNA markers

Genetic differentiation among the giant panda populations was estimated by the *D*_est_ values for *Aime*-MHC and mtDNA. At the MHC loci, the overall genetic differentiation *D*_est_ estimates were 0.198 (for the arithmetic mean) and 0.116 (for the harmonic mean) for all loci across populations. The pairwise *D*_est_ estimates between populations ranged from 0.033 to 0.353 (for the arithmetic mean) and from 0.011 to 0.179 (for the harmonic mean; Table S5). For the mtDNA, the overall genetic differentiation *D*_est_ value was 0.221 across all panda populations, while the pairwise estimate between populations varied from −0.008 to 0.411 (Table S5). Significant associations between geographic and genetic distance were identified from both the arithmetic and the harmonic mean of *D*_est_ at MHC loci by Mantel tests (*P* = 0.013 and *P* = 0.011, respectively), but no isolation by distance was detected in the mtDNA (*P*>0.1).

### Positive selection of the Aime-MHC class II loci

Higher non-synonymous (*d*_N_) than synonymous (*d*_S_) substitutions among the MHC sequences is inferred as indicating positive selection, whereas a lower proportion of non-synonymous substitutions is evidence for purifying selection in a population [18]. The ratios of nonsynonymous (*d*_N_) and synonymous (*d*_S_) substitutions at the *Aime*-MHC class II loci differed (Table 3). Ratios of *d*_N_/*d*_S_ greater than 1 were detected at the ABS sites of 3 beta loci (3.500 for *Aime-*DQB1, 4.950 for *Aime-*DQB2, and 2.176 for *Aime-*DRB3), providing evidence for positive selection in the giant panda (Table 3). Z-tests provided significant support for hypotheses of positive selection at *Aime-*DQB1 (*P* = 0.004) and *Aime-*DQB2 (*P* = 0.001), but not at DRB3 (*P* = 0.066). In addition, an excess of non-synonymous substitutions was identified in all sites at DQB2, (*P*<0.05; Table 3), indicating extensive positive selection throughout exon2 of this gene. Within the beta loci, evidence for strong positive selection was detected at DQB1 and DQB2 in the XXL population, in which significantly higher *d*_N_/*d*_S_ ratios were observed in the ABS and across all sites (Table S6). Although not significant, higher *d*_N_/*d*_S_ ratios were consistently observed in the ABS sites of the 3 alpha loci (Table 3). When differences in *d*_N_/*d*_S_ at the non-ABS sites were analyzed, non-synonymous substitutions were consistently lower than or similar to synonymous substitutions for all 6 loci (Table 3), indicating that there was a trend towards purifying selection in the non-ABS regions of the *Aime*-MHC genes. We compared the *d*_N_/*d*_S_ values of the giant panda with those of the brown bear and black bear. In the brown bear [54], the *d*_N_/*d*_S_ values in the ABS sites at DQB and DQA were lower than those of the giant panda (DQB: 2.750 vs. 3.500, 2.750 vs. 4.950; DQA: 1.225 vs. 1.261, 1.225 vs. 1.359). However, the *d*_N_/*d*_S_ value in the ABS of DRB was higher than that of the giant panda DRB3 (5.084 vs. 2.176). In the black bear [58], the *d*_N_/*d*_S_ value was also lower than that of *Aime-*DQB1 (1.48 vs. 3.500).

**Table 3:** Synonymous (*d*_S_) and nonsynonymous (*d*_N_) substitutions for the *Aime*-MHC class II genes.

Locus		*d*_N_	*d*_S_	*d*_N_/*d*_S_	*P*
DQA1	ABS	0.116±0.048	0.092±0.063	1.261	0.792
	non-ABS	0.010±0.006	0.015±0.010	0.667	0.731
	ALL	0.031±0.012	0.027±0.013	1.148	0.778
DQA2	ABS	0.125±0.044	0.092±0.059	1.359	0.688
	non-ABS	0.017±0.007	0.019±0.013	0.895	0.898
	ALL	0.038±0.011	0.030±0.014	1.267	0.654
DRA	ABS	0.022±0.022	0.000±0.000	–	0.294
	non-ABS	0.007±0.007	0.039±0.027	0.179	0.284
	ALL	0.011±0.008	0.030±0.021	0.367	0.412
DQB1	ABS	0.196±0.051	0.056±0.042	3.500	**0.004**
	non-ABS	0.027±0.008	0.028±0.013	0.964	0.921
	ALL	0.064±0.015	0.034±0.016	1.882	0.041
DQB2	ABS	0.297±0.063	0.060±0.044	4.950	**0.001**
	non-ABS	0.046±0.017	0.040±0.019	1.150	0.785
	ALL	0.102±0.022	0.044±0.018	2.318	0.025
DRB3	ABS	0.235±0.037	0.108±0.037	2.176	0.066
	non-ABS	0.028±0.008	0.037±0.011	0.757	0.503
	ALL	0.072±0.0140	0.052±0.012	1.385	0.269

Standard errors (in parentheses) were obtained through 1000 bootstrap replicates.

*P* values in bold indicate *d*_N_ is significantly larger than *d*_S_ (*P*<0.05).

We also checked for the signature of positive selection using PAML (Table 4). The likelihood-ratio test (M7 vs. M8) showed significant *P* values at all *Aime*-MHC class II loci except for *Aime*-DRA (Table S7), suggesting positive selection. In the brown bear [54], there was evidence of positive selection at DRB with an ω estimate of 12.181 (giant panda DRB3, ω = 3.081); however, no signal positive selection was detected at DQB, which is the opposite of what was observed in the giant panda DQB loci (ω = 15.996 for DQB1, ω = 10.250 for DQB2). In the black bear [58], the ω estimate of 5.71 at DQB was lower than that of the giant panda. Bayesian analysis identified different numbers of codons under positive selection at *Aime*-MHC class II loci (6 for DQA1, 4 for DQA2, 6 for DQB1, 7 for DQB2, and 7 for DRB3).

**Table 4:** Inference of positive selection for alpha and beta genes in giant panda with different models.

Locus	Model	InL	Parameters	Positively selected sites
DQA1	M0 (one ratio)	−537.489	ω = 1.080	
	M7 (nearly neutral with beta)	−522.272	p = 0.005, q = 0.047	
	M8 (beta & ω)	−499.021	p_0_ = 0.985, p = 0.005, q = 0.045, ω2 = 28.410	23**, **32** **, **53** *, 54*, **67** **, **77** *
DQA2	M0 (one ratio)	−523.760	ω = 1.229	
	M7 (nearly neutral with beta)	−519.361	p = 0.005, q = 0.012	
	M8 (beta & ω)	−501.255	p_0_ = 0.995, p = 99.000, q = 71.048, ω2 = 58.615	23**, **32** **, **67** **
DRA	M0 (one ratio)	−343.406	ω = 0.382	
	M7 (nearly neutral with beta)	−343.406	p = 61.310, q = 99.000	
	M8 (beta & ω)	−343.406	p_0_ = 0.100, p = 61.309, q = 99.000, ω2 = 1.000	
DQB1	M0 (one ratio)	−783.408	ω = 0.541	
	M7 (nearly neutral with beta)	−772.205	p = 1.120, q = 1.138	
	M8 (beta & ω)	−720.368	p_0_ = 0.995, p = 0.011, q = 0.039, ω2 = 15.996	**26** **, **28** **, **47** **, 56*, **57** **, **61** **
DQB2	M0 (one ratio)	−515.205	ω = 0.698	
	M7 (nearly neutral with beta)	−507.847	p = 0.005, q = 0.012	
	M8 (beta & ω)	−502.949	p_0_ = 0.973, p = 0.005, q = 0.011, ω2 = 10.250	**9** *, **26** *, **28** *, 42*, 56*, **57** **, **61** *
DRB3	M0 (one ratio)	−756.906	ω = 0.335	
	M7 (nearly neutral with beta)	−732.770	p = 0.005, q = 0.020	
	M8 (beta & ω)	−728.612	p_0_ = 0.946, p = 6.933, q = 71.662, ω2 = 3.081	**11** *, **26** **, **57** *, **61** **, **67** *, **71** **, 94*

Note: ω =  *d*_N_/*d*_S_; p and q: parameters of beta distribution;

p_0_: proportion of sites with ω≤1;

ω2: value of ω for sites under positive selection.

*: posterior probability, *P*<0.95.

**: posterior probability, *P*<0.99.

Figures in bold are referred to as ABS sites.

### Allelic relationships of the Aime-MHC class II loci

**Figure 4:**
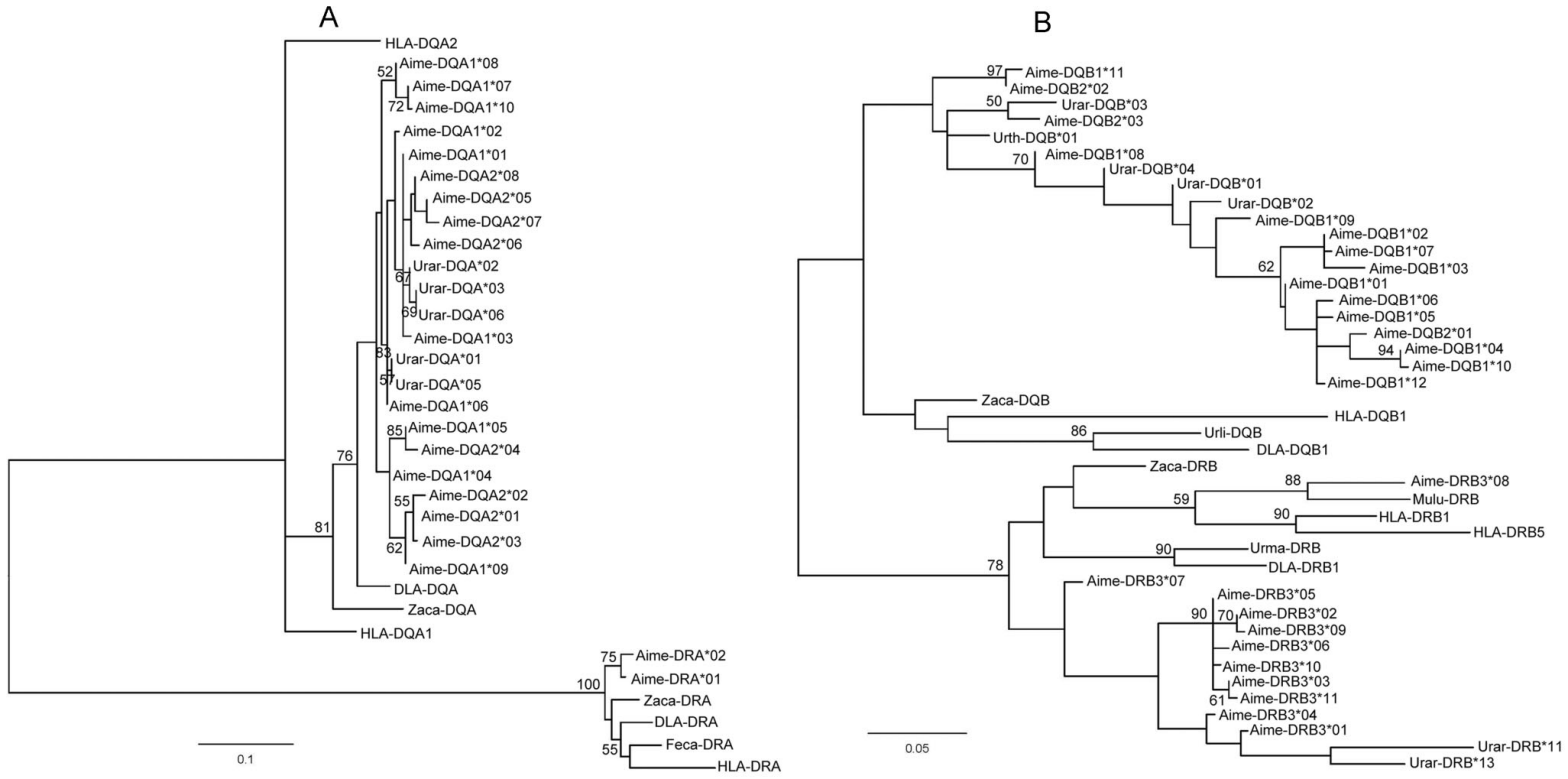
Maximum likelihood phylogenetic trees. Maximum likelihood (ML) phylogenetic relationships of the *Aime*-MHC class II alpha (A) and beta (B) alleles. Bootstrap values less than 50 (50%) are not shown.

In the ML trees constructed for the *Aime* MHC alpha and beta genes, most alleles from the giant panda were more closely related to each other than to those from other carnivores (Figure 4). In the alpha tree, all of the DQA1 and DQA2 alleles formed a single clade along with 5 alleles from the brown bear (Urar-DQA*01, 02, 03, 05, and 06). In the DQB lineage, the DQB sequence of the Asian black bear was clustered in the clade of panda DQB alleles by a bootstrap value lower than 50% (Figure 4B). Three DQB sequences of the brown bear (Urar-DQB*01, 02, and 04) were clustered in the clade of panda DQB alleles by a bootstrap value of 70%. Five DQB alleles detected only in the XXL population (DQB1*08, 09, and 11, and DQB2*02 and 03), which diverged greatly from the other DQB alleles (Figure S1B in Supporting information), were located basally in the *Aime-*DQB lineage, indicating their ancient status among the panda alleles. In the DRB3 lineage, the *Aime-*DRB3*08 allele was clustered with the *Mulu*-DRB allele from the European mink by a bootstrap value of 88%, which indicated the trans-species polymorphism of the *Aime-*MHC. In the DQB lineage, the *Aime*-DQB2*03 allele was clustered with the Urar-DQB*03 allele from the brown bear; however, this allele was cluster by a low bootstrap value of 50%; therefore, we could not draw a conclusion of trans-species polymorphism.

## Discussion

### The diversity center/refuge of the giant panda

The Qinling and Sichuan subspecies live in the Qinling and Hengduan Mountains, respectively, and the topography and climate of these 2 habitats are quite different. The DNA fingerprint network relationships showed that the Qinling and Sichuan subspecies formed separate clades (Figure 3), which supports the notion that these 2 subspecies have independent population histories [17]. The observed mismatch distribution and Bayesian skyline plot analysis of the mtDNA haplotypes for the Sichuan and Qinling subspecies corresponded to the characteristics of expanding and stable populations (Figure 2A), respectively. However, the mtDNA-based network did not indicate obvious separate clades for the 2 subspecies, and this may have resulted from female-biased dispersal patterns in the giant panda, which lead to lower differentiation at maternally-inherited mtDNA than expected. In addition, comparisons of MHC divergence revealed that there were specific alleles in each of the 2 subspecies (Qinling subspecies: DQA2*04 and Sichuan subspecies: DQB1*07). These findings support the idea that the Qinling and Sichuan subspecies have different evolutionary histories, and indicate that these 2 subspecies likely have different diversity centers. Previous studies using neutral markers revealed that the relatively small Qinling population possessed high levels of genetic variation similar to those of the 2 large Sichuan populations (MSH and QLA) [13], [16], suggesting that QLI has a different diversity center from the Sichuan populations. Furthermore, the presence of a 6.9-kb restriction fragment specific to the Sichuan populations and the absence of a fragment specific to QLI seem to indicate that the last bottleneck split the giant panda species into a relatively large ancestral QLI population and a small original population that became the Sichuan subspecies [16]. This, combined with the independent phylogeography of the Qinling subspecies, further supports the idea that this subspecies was QLI-derived, but the Sichuan subspecies originated from an unknown center.

In this study, the DNA fingerprint network relationships showed that all of the XXL individuals formed the center of the network within the Sichuan subspecies, whereas individuals from other populations formed the tips (Figure 3), which suggests that this population has an ancestral status at the center of the network [59], and indicates that the XXL population represents a refuge for the Sichuan subspecies after the split of the species. Nevertheless, this result was inconsistent with DNA fingerprint MP analysis, and it may be due to DNA fingerprinting of a dominant marker that was not sensitive in the phylogenetic analysis. As the giant panda populations were deduced to have differentiated into 2 subspecies 10,000 years ago [16], which is congruent with the most recent ice age in Western China [60], [61], we inferred that XXL represents a glacial refuge of the giant panda. Comparisons across the 6 panda populations (Sample size: QLI = 64, MSH = 41, QLA = 49, DXL = 16, XXL = 33, and LSH = 40) revealed that the XXL population had the most MHC alleles (35 alleles, 9 were unique), making it a diversity center of the giant panda. This was in good agreement with a previous study that used 9 microsatellite markers to show that the XXL population had the highest level of allelic richness [9]. The XXL population, which is currently 1 of the smallest and most fragmented populations, experienced a drastic population reduction (60-fold) approximately 250 years ago [15], which suggests that the population should have undergone genetic drift and a rapid loss of genetic diversity [12]. However, contrary to this expectation, XXL was found to have the highest level of genetic variability. Among the MHC class II genes, we observed that diversity tended to decline in both allele number and heterozygosity of the XXL population compared to the diversification pattern of the other 4 Sichuan populations (LSH-DXL-QLA-MSH; Tables 1 and 2). This observation is in accordance with the characteristics of refuges that have experienced ice ages [62]. In the lineage based on the *Aime*-MHC sequences, 5 unique DQB alleles were basal within the DQB lineages (Figure 4B), indicating that these sequences had ancient origins in this population. Furthermore, the DQB2*02 and DQB2*03 alleles, which were found only in XXL, diverged significantly from the widely-distributed DQB2*01 allele in exon2 (Figure S1B in Supporting Information) and intron1 (data not shown). These divergent alleles could not be generated by a few step-wise mutations in the near past, further supporting the ancient diversity of the XXL population. These results are consistent with our inference that the extant giant panda Sichuan subspecies may have expanded from an ancestral XXL population.

### Diversity patterns in *Aime*-MHC class II genes

The MHC, which is one of the most polymorphic regions in vertebrates, plays significant roles in adaptive immunity [18]. However, this is the first time adaptive variations at MHC loci have been investigated in extant wild panda populations. Six functional MHC genes were genotyped, and a total of 46 MHC alleles, including 19 novel alleles, were identified in the 6 giant panda populations, supporting the assertion that this rare species maintains relatively abundant variations in its adaptive immune system [30].

In the *Aime*-MHC, we found that the *Aime*-DQ genes had unusual diversity patterns. First, most mammals have more variations at DRB loci than at DQB loci, and more variations in beta genes than in alpha genes. For example, the human HLA has 873, 144, and 35 alleles at the DRB1, DQB1, and DQA1 loci, respectively; the dog DLA has 52, 36, and 16 alleles at the DRB1, DQB1, and DQA1 loci, respectively; and cattle have 120, 74, and 51 alleles at the BoLA-DRB3, DQB, and DQA loci, respectively (http://www.ebi.ac.uk); the brown bear, a close relative of the giant panda, has 31, 4, and 5 alleles at the DRB, DQB, and DQA loci, respectively [54]. In contrast, we found that the giant panda had 12 alleles at DQB1, 11 at DRB3, and 10 at DQA1. Including the alleles from DQA2 and DQB2, the giant panda has many more DQ alleles than DR alleles (33∶13). In addition, we identified 9 alleles unique to individuals from the XXL refuge: DQB1*08–*12, DQB2*02–*03, and DRB3*10–*11 (Table 2). Seven of these were derived from the DQB genes. This indicates the special characteristics of the DQB alleles in the XXL refuge. Our previous study revealed that the giant panda has numerous DQ genes, many more than those found in other carnivores (e.g., the dog and cat). Numerous DQ genes are more commonly found in herbivores [29]. Moreover, frequent recombination was detected in the *Aime*-DQ sub-region, which lead to the allelic polymorphisms of *Aime*-MHC genes [30]. While different species adopt distinct evolutionary strategies in MHC class II genes to cope with pathogens [63], the above findings collectively indicate that the giant panda developed its adaptive strategies by means of DQ subregion expansion. The high level of allelic polymorphisms of the DQ genes, and the bias for DQ diversity is likely important for the adaptation of this carnivore.

### Habitat fragmentation and gene flow

Historically, we believe that habitat fragmentation during the ice age shaped the 2 refuges (QLI and XXL) and lead to the development of the Qinling and Sichuan subspecies. The subsequent expansion of the Sichuan subspecies can be seen from the inference of contiguous range expansion by the bell-shaped, mismatch distribution and Bayesian skyline plot analysis of the mtDNA haplotypes (Figure 2A). However, our analysis also suggested the possibility that there was a second contact between the Qinling and Sichuan subspecies. The H8, H10, and H12 haplotypes are shared by the Qinling and Sichuan pandas, indicating past inter-subspecies gene flow, while the H2, H3, H6, H7, H8, and H21 haplotypes were distributed among the different Sichuan populations, indicating intra-subspecies gene exchanges (Figure 1 and Table S3 in Supporting Information). A previous study showed that the giant panda exhibits female-biased dispersal [64], which may suggest why the maternally-inherited mtDNA-based network did not suggest obvious separate clades from the 2 subspecies as expected. However, only 8 of the 43 mtDNA haplotypes were shared among different populations (Table S3 in Supporting Information), which is in good agreement with the restricted gene flow detected for the giant panda revealed by microsatellites [9], [13] and DNA fingerprinting [16]. In addition, although 35 of 46 alleles at functional *Aime-*MHC loci were shared among the populations (Tables 1 and 2), the isolation by distance pattern of the MHC loci was proved by the Mantel tests of pairwise *D*_est_ in this study.

### Evidence of balancing selection at *Aime*-MHC genes

A higher rate of substitutions at non-synonymous sites relative to synonymous positions often results from balancing selection [65]. Here, higher *d*_N_/*d*_S_ ratios were observed in the ABS of 6 *Aime-*MHC alpha and beta genes (Table 3), which is in accordance with published data on beta genes in bears [54], [58], [66] and other mammals [67], [68].

Trans-species polymorphism, where similar alleles are found in related species due to the passage of alleles from ancestral to descendant species, is hypothesized to be maintained by balancing selection [69]. Here, we showed evidence of trans-species polymorphism in the giant panda MHC sequences at DRB. Trans-species polymorphism has been previously reported in the DRB lineages of Ursidae species [54], [58]. Although we did not find evidence for mixing of the DRB alleles of the giant panda with those of the bear lineage, the DQA and DQB sequences in the present study were found to intermingle with alleles from brown and black bears, respectively. The clustering of *Aime-*DRB3*08 with the DRB allele of the European mink indicates that these likely represent sequences that originated from a very distant common ancestor, and have either been lost or have not yet been detected in other related species.

Therefore, our findings collectively indicate that balancing selection maintained abundant variations in the adaptive immune system of the giant panda.

## Supporting Information

Figure S1 **Sequence alignments.** Multiple sequence alignments of the predicted amino acid sequences deduced from the *Aime*-MHC class II alpha (A) and beta (B) genes. Sequences that are newly reported in this paper are shaded. Dots indicate identity with the first sequence, while dashes represent amino acid deletions. Plus symbols under the alignment indicate amino acids that are predicted to be involved in antigen binding based on comparison to the corresponding HLA sequences [20]. The HLA alpha and beta genes used as reference sequences were HLA-DQA1 (DQ284439), HLA-DRA (NM_019111), HLA-DQB1 (AM259941), and HLA-DRB3 (NM_022555).
(TIF)

Table S1 **Sampling information for the giant pandas analyzed in this study.**
(DOC)

Table S2 **Primer sets used to amplify the entirety of exon2 from the six** ***Aime-*** **MHC class II genes and a partial sequence of the mitochondrial control region.**
(DOC)

Table S3 **Distribution and frequency of the mtDNA haplotypes among the six giant panda populations.**
(DOC)

Table S4 **Sequence divergence among the six** ***Aime*** **-MHC class II genes.**
(DOC)

Table S5 **Pairwise** ***D*** **_est_ estimates for the** ***Aime*** **-MHC class II loci (lower diagonal) and mtDNA (upper diagonal) among the six giant panda populations.**
(DOC)

Table S6 **Synonymous (** ***d*** **_S_) and nonsynonymous (** ***d*** **_N_) substitutions for the Aime-MHC beta genes in each population.** *P* is the significance of the difference between *d*_N_ and *d*_S_ in the test of positive selection.
(DOC)

Table S7 **The likelihood ratio test of positive selection for the giant panda MHC genes.**
(DOC)
