# Supplementary figures and images for: Patterns of Adaptive and Neutral Diversity Identify the Xiaoxiangling Mountains as a Refuge for the Giant Panda
Source: PLoS One. 2013 Jul 19;8(7):e70229. doi: 10.1371/journal.pone.0070229 (PMC3716684; doi:10.1371/journal.pone.0070229)

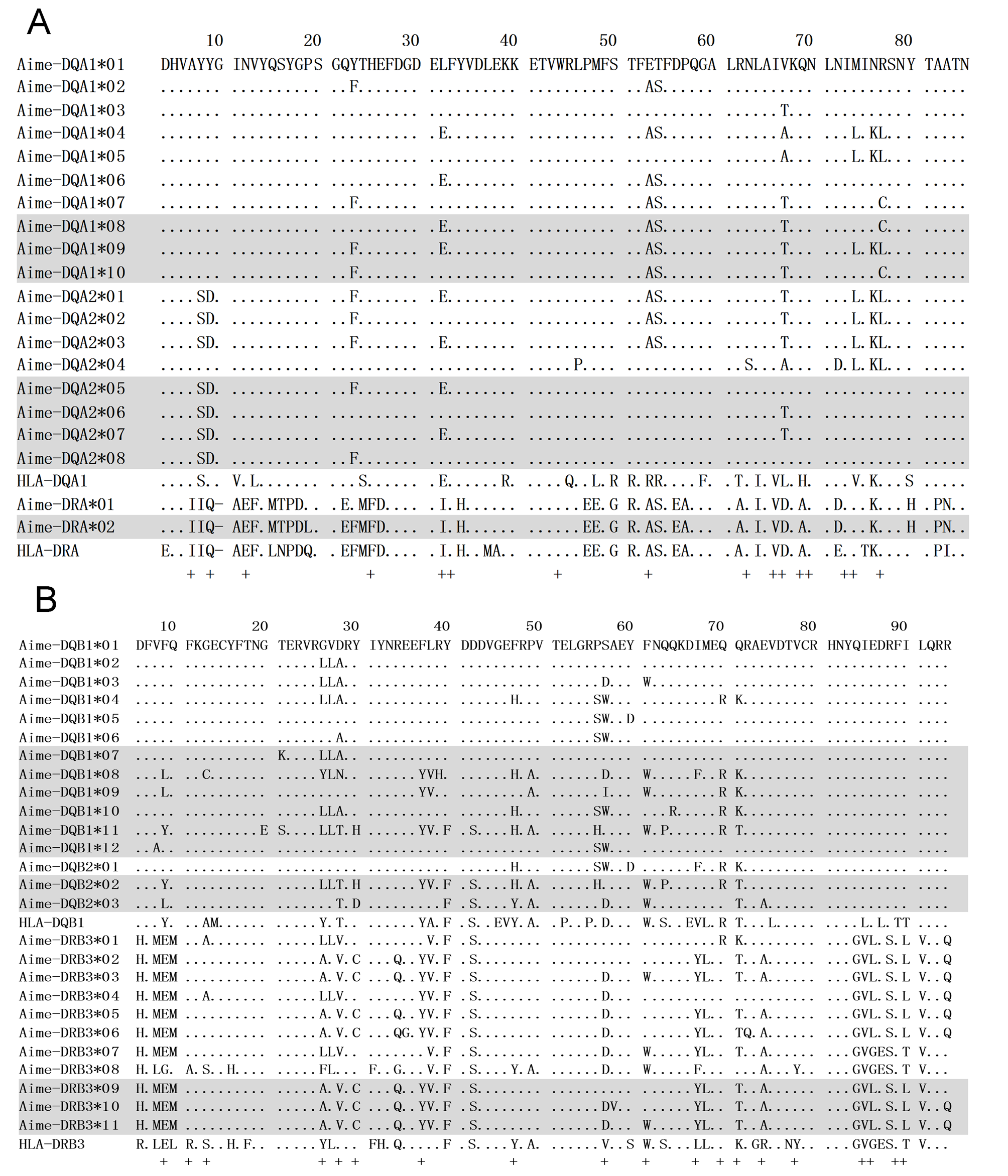

Supplement: Figure S1 — Sequence alignments. Multiple sequence alignments of the predicted amino acid sequences deduced from the Aime-MHC class II alpha (A) and beta (B) genes. Sequences that are newly reported in this paper are shaded. Dots indicate identity with the first sequence, while dashes represent amino acid deletions. Plus symbols under the alignment indicate amino acids that are predicted to be involved in antigen binding based on comparison to the corresponding HLA sequences [20]. The HLA alpha and beta genes used as reference sequences were HLA-DQA1 (DQ284439), HLA-DRA (NM_019111), HLA-DQB1 (AM259941), and HLA-DRB3 (NM_022555). (TIF) [file pone.0070229.s001.tif]
