# Supplementary material for: Patterns of Adaptive and Neutral Diversity Identify the Xiaoxiangling Mountains as a Refuge for the Giant Panda
Source: PLoS One. 2013 Jul 19;8(7):e70229. doi: 10.1371/journal.pone.0070229 (PMC3716684; doi:10.1371/journal.pone.0070229)
Supplement: Table S1 — Sampling information for the giant pandas analyzed in this study. (DOC) [file pone.0070229.s002.doc]

Table S1 Sampling information for the giant pandas analyzed in this study.

| Subspecies | ^a^ Population | ^b^ Sample no. | Material type | Sampling year |
| --- | --- | --- | --- | --- |
| Qinling subspecies | Qinling | QLI1 | blood | 2007 |
| (*A. m. qinlingensis*) | QLI, n=**64** | QLI2-QLI3 | blood | 2009 |
|  |  | QLI4- QLI52 | skin | 1990-2008 |
|  |  | QLI53-QLI64 | feces | 2008 |
| Sichuan subspecies | Minshan | MSH1-MSH8 | blood | 2007-2009 |
| (*A.m. melanoleuca*) | MSH, n=**41** | MSH9 | skin | 1976 |
|  |  | MSH10-MSH39 | skin | 1988-2003 |
|  |  | MSH40 | feces | 2007 |
|  |  | MSH41 | feces | 2009 |
|  | Qionglai | QLA1-QLA19 | blood | 2007-2009 |
|  | QLA, n=**49** | QLA20 | liver | 1995 |
|  |  | QLA21-QLA22 | liver | 2008 |
|  |  | QLA23 | skin | 1980 |
|  |  | QLA24 | skin | 1989 |
|  |  | QLA25-QLA49 | skin | 1991-2005 |
|  | Daxiangling | DXL1-25 (*16*) | feces | 2009 |
|  | DXL, n=**16** |  |  |  |
|  | Xiaoxiangling | XXL1 | blood | 2007 |
|  | XXL, n=**33** | XXL2 | skin | 1995 |
|  |  | XXL3-XXL53 (*31*) | feces | 2009 |
|  | Liangshang | LSH1-3 | blood | 2007 |
|  | LSH, n=**40** | LSH4 | skin | 1982 |
|  |  | LSH5-LSH13 | skin | 1992-2000 |
|  |  | LSH14-LSH60 (*27*) | feces | 2009 |

^a^ Numbers in bold indicates sample size. ^b^ Numbers in italic are the numbers of individuals represented the wild-sampled fecal samples of the DXL, XXL and LSH populations, as confirmed based on multiple-loci MHC genotyping plus sampling information.
