# Supplementary material for: Patterns of Adaptive and Neutral Diversity Identify the Xiaoxiangling Mountains as a Refuge for the Giant Panda
Source: PLoS One. 2013 Jul 19;8(7):e70229. doi: 10.1371/journal.pone.0070229 (PMC3716684; doi:10.1371/journal.pone.0070229)
Supplement: Table S2 — Primer sets used to amplify the entirety of exon2 from the six Aime- MHC class II genes and a partial sequence of the mitochondrial control region. (DOC) [file pone.0070229.s003.doc]

Table S2 Primer sets used to amplify the entirety of exon2 from the six Aime-MHC class II genes and a partial sequence of the mitochondrial control region.

| Locus | Primer name | Primer sequence (5’→3’) | Size (bp) | Ta (ºC) |
| --- | --- | --- | --- | --- |
| DRA | RAF | TTCCTCTCCCCCTCCTGGTTCC | 330 | 55 |
|  | RAR | TAGGATTCCCGTGTCTAGGAGTGC |  |  |
| DRB3 | **DRB1F3** | **AGCGGATCGTCTCTGTCCCCA** | 393 | 57 |
|  | **DRB13DN3** | **CCCTGTATGTTCACTCCGGATGT** |  |  |
| DQA1 | **QA1UP3B** | **GTTTAGTAATCATGCTTTCTCCC** | 332 | 59 |
|  | DQA1DN2 | AGAGGCAGAGCATTGGACACATAC |  |  |
| DQA2 | DQA2UP1 | GTTTCTTCCGTCACTTGGCTTAATAAGG | 349 | 63 |
|  | DQA2DN1 | AGGCAGAGCATTGGACACATACCAT |  |  |
| DQB1 | DQBUP1 | AGTGACCCGCGGTGATTCCC | 379 | 59 |
|  | **DQB1R6** | **CCAGCCGGCTGGGTCCCG** |  |  |
| DQB2 | DQBUP1 | AGTGACCCGCGGTGATTCCC | 390 | 59 |
|  | **DQB2R5** | **CCCCGCGTCCCCGCCCCTCC** |  |  |
| control | **CRF1** | **AACAACCCCACTACCAGCAC** | 706-708 | 52 |
| region | **CRR1** | **CATTGACTGAATTGCACCTT** |  |  |

The bolded primers were designed in this study while the others were derived from Chen *et al*. [1].

References:

[1] Chen YY, Zhang YY, Zhang HM, Ge YF, Wan QH, et al. (2010) Natural Selection Coupled With 540 Intragenic Recombination Shapes Diversity Patterns in the Major Histocompatibility Complex 541 Class II Genes of the Giant Panda. J Exp Zool B Mol Dev Evol 314B: 208–223
