# Supplementary material for: Patterns of Adaptive and Neutral Diversity Identify the Xiaoxiangling Mountains as a Refuge for the Giant Panda
Source: PLoS One. 2013 Jul 19;8(7):e70229. doi: 10.1371/journal.pone.0070229 (PMC3716684; doi:10.1371/journal.pone.0070229)
Supplement: Table S3 — Distribution and frequency of the mtDNA haplotypes among the six giant panda populations. (DOC) [file pone.0070229.s004.doc]

Table S3 Distribution and frequency of the mtDNA haplotypes among the six giant panda populations.

| haplotype | population | | | | | | haplotype | population | | | | | |
| --- | --- | --- | --- | --- | --- | --- | --- | --- | --- | --- | --- | --- | --- |
|  | QLA | MSH | LSH | DXL | XXL | QLI |  | QLA | MSH | LSH | DXL | XXL | QLI |
| HP1 | 1 |  |  |  |  |  | HP22 |  |  | 1 |  |  |  |
| HP2 | 3 | 2 |  |  |  |  | HP23 |  |  | 1 |  |  |  |
| HP3 | 9 | 2 |  | 4 |  |  | HP24 |  |  | 1 |  |  |  |
| HP4 | 1 |  |  |  |  |  | HP25 |  |  | 1 |  |  |  |
| HP5 | 1 |  |  |  |  |  | HP26 |  |  |  | 6 |  |  |
| HP6 | 6 | 7 | 1 |  | 6 |  | HP27 |  |  |  | 1 |  |  |
| HP7 | 1 | 2 |  |  |  |  | HP28 |  |  |  | 1 |  |  |
| HP8 | 3 |  | 6 |  | 8 | 1 | HP29 |  |  |  | 1 |  |  |
| HP9 | 1 |  |  |  |  |  | HP30 |  |  |  | 1 |  |  |
| HP10 | 1 |  |  |  |  | 8 | HP31 |  |  |  |  | 1 |  |
| HP11 |  |  |  |  | 9 |  | HP32 |  |  |  |  | 1 |  |
| HP12 |  | 2 |  |  |  | 3 | HP33 |  |  |  |  | 2 |  |
| HP13 |  | 1 |  |  |  |  | HP34 |  |  |  |  | 1 |  |
| HP14 |  | 3 |  |  |  |  | HP35 |  |  |  |  | 1 |  |
| HP15 |  | 1 |  |  |  |  | HP36 |  |  |  |  |  | 4 |
| HP16 |  | 1 |  |  |  |  | HP37 |  |  |  |  |  | 1 |
| HP17 |  | 2 |  |  |  |  | HP38 |  |  |  |  |  | 1 |
| HP18 |  |  | 3 |  |  |  | HP39 |  |  |  |  |  | 1 |
| HP19 |  |  | 5 |  |  |  | HP40 |  |  |  |  |  | 2 |
| HP20 |  |  | 6 |  |  |  | HP41 |  |  |  |  |  | 1 |
| HP21 |  |  | 3 | 2 |  |  | HP42 |  |  |  |  |  | 3 |
|  |  |  |  |  |  |  | HP43 |  |  |  |  |  | 1 |
