# Supplementary material for: Patterns of Adaptive and Neutral Diversity Identify the Xiaoxiangling Mountains as a Refuge for the Giant Panda
Source: PLoS One. 2013 Jul 19;8(7):e70229. doi: 10.1371/journal.pone.0070229 (PMC3716684; doi:10.1371/journal.pone.0070229)
Supplement: Table S4 — Sequence divergence among the six Aime -MHC class II genes. (DOC) [file pone.0070229.s005.doc]

Table S4 Sequence divergence among the six *Aime*-MHC class II genes.

| category of *Aime*-MHC II class genes | locus | number of alleles | number of variable sites | |
| --- | --- | --- | --- | --- |
|  |  |  | bp (range) | aa ^a^ (range) |
| alpha (DQA: 249 bp ; DRA: 246 bp) | DQA1 | 10 | 15 (1-14) | 8 (0-8) |
|  | DQA2 | 8 | 21 (1-17) | 13 (0-11) |
|  | DRA | 2 | 4 (4) | 2 (2) |
|  | Alpha | 20 | 99 (1-92) | 40 (0-36) |
| beta (270 bp) | DQB1 | 12 | 43 (1-32) | 25 (1-20) |
|  | DQB2 | 3 | 33 (19-27) | 20 (12-17) |
|  | DRB3 | 11 | 61 (1-53) | 30 (0-25) |
|  | Beta | 26 | 94 (1-61) | 45 (0-30) |

^a^ Point mutations were observed between seven pairs of sequences across four loci. There were synonymous substitutions between DQA1*07 and DQA1*10, DQA2*01 and DQA2*03, DRB3*03 and DRB3*11, and non-synonymous substitutions between DQB1*02 and DQB1*07, DQB1*04 and DQB1*10, DRB3*02 and DRB3*09, DRB3*05 and DRB3*10.
