# Supplementary material for: Patterns of Adaptive and Neutral Diversity Identify the Xiaoxiangling Mountains as a Refuge for the Giant Panda
Source: PLoS One. 2013 Jul 19;8(7):e70229. doi: 10.1371/journal.pone.0070229 (PMC3716684; doi:10.1371/journal.pone.0070229)
Supplement: Table S5 — Pairwise D est estimates for the Aime -MHC class II loci (lower diagonal) and mtDNA (upper diagonal) among the six giant panda populations. (DOC) [file pone.0070229.s006.doc]

Table S5 Pairwise *D*_est_ estimates for the *Aime*-MHC class II loci (lower diagonal) and mtDNA (upper diagonal) among the six giant panda populations.

|  | QLI | MSH | QLA | LSH | DXL | XXL |
| --- | --- | --- | --- | --- | --- | --- |
| QLI | - | -0.008 | 0.259 | 0.113 | 0.211 | 0.032 |
| MSH | 0.142, **0.072** | - | 0.222 | 0.259 | 0.291 | 0.034 |
| QLA | 0.182, **0.107** | 0.141, **0.054** | - | 0.351 | 0.365 | 0.242 |
| LSH | 0.250, **0.150** | 0.193, **0.080** | 0.060, **0.019** | - | 0.176 | 0.210 |
| DXL | 0.353, **0.166** | 0.324, **0.159** | 0.190, **0.078** | 0.142, **0.053** | - | 0.411 |
| XXL | 0.315, **0.179** | 0.227, **0.101** | 0.096, **0.038** | 0.033, **0.011** | 0.176, **0.063** | - |

For *Aime*-MHC, estimates of both the arithmetic and harmonic (in bold) means of *D*_est_ were listed. A negative *D*_est_ is considered to be not significantly different from zero.
