# Supplementary material for: Patterns of Adaptive and Neutral Diversity Identify the Xiaoxiangling Mountains as a Refuge for the Giant Panda
Source: PLoS One. 2013 Jul 19;8(7):e70229. doi: 10.1371/journal.pone.0070229 (PMC3716684; doi:10.1371/journal.pone.0070229)
Supplement: Table S6 — Synonymous ( d S) and nonsynonymous ( d N) substitutions for the Aime-MHC beta genes in each population. P is the significance of the difference between d N and d S in the test of positive selection. (DOC) [file pone.0070229.s007.doc]

Table S6 Synonymous (*d*_S_) and nonsynonymous (*d*_N_) substitutions for the Aime-MHC beta genes in each population. *P* is the significance of the difference between *d*_N_ and *d*_S_ in the test of positive selection.

| Population | Values | DQB1 | | | DQB2a | | | DRB3 | | |
| --- | --- | --- | --- | --- | --- | --- | --- | --- | --- | --- |
|  |  | ABS | Non-ABS | All | ABS | Non-ABS | All | ABS | Non-ABS | All |
| QLI | *d*_N_ | 0.120±0.045 | 0.125±0.007 | 0.036±0.014 | - | - | - | 0.270±0.041 | 0.031±0.009 | 0.082±0.015 |
|  | *d*_S_ | 0.065±0.046 | 0.019±0.013 | 0.028±0.014 | - | - | - | 0.128±0.040 | 0.041±0.013 | 0.060±0.014 |
|  | *P* | 0.099 | 0.677 | 0.544 | - | - | - | 0.051 | 0.517 | 0.297 |
| MSH | *d*_N_ | 0.128±0.055 | 0.012±0.006 | 0.036±0.012 | - | - | - | 0.314±0.044 | 0.033±0.010 | 0.092±0.017 |
|  | *d*_S_ | 0.055±0.043 | 0.026±0.014 | 0.031±0.014 | - | - | - | 0.156±0.049 | 0.050±0.014 | 0.073±0.017 |
|  | *P* | 0.043 | 0.303 | 0.707 | - | - | - | 0.054 | 0.313 | 0.456 |
| QLA | *d*_N_ | 0.112±0.040 | 0.012±0.006 | 0.034±0.011 | - | - | - | 0.293±0.043 | 0.031±0.010 | 0.086±0.017 |
|  | *d*_S_ | 0.054±0.037 | 0.026±0.013 | 0.031±0.013 | - | - | - | 0.141±0.045 | 0.045±0.014 | 0.066±0.015 |
|  | *P* | 0.034 | 0.325 | 0.834 | - | - | - | 0.065 | 0.376 | 0.384 |
| DXL | *d*_N_ | 0.000±0.000 | 0.007±0.007 | 0.005±0.005 | - | - | - | 0.154±0.038 | 0.013±0.006 | 0.042±0.011 |
|  | *d*_S_ | 0.000±0.000 | 0.000±0.000 | 0.000±0.000 | - | - | - | 0.022±0.021 | 0.010±0.001 | 0.013±0.009 |
|  | *P* | 1.000 | 0.319 | 0.295 | - | - | - | 0.006 | 0.768 | 0.023 |
| XXL | *d*_N_ | 0.251±0.085 | 0.028±0.008 | 0.070±0.017 | 0.297±0.063 | 0.046±0.017 | 0.102±0.022 | 0.234±0.034 | 0.028±0.008 | 0.072±0.014 |
|  | *d*_S_ | 0.058±0.048 | 0.030±0.013 | 0.035±0.014 | 0.060±0.044 | 0.040±0.019 | 0.044±0.018 | 0.113±0.034 | 0.046±0.013 | 0.060±0.013 |
|  | *P* | 0.005 | 0.883 | 0.050 | 0.001 | 0.785 | 0.025 | 0.084 | 0.279 | 0.560 |
| LSH | *d*_N_ | 0.119±0.043 | 0.012±0.006 | 0.036±0.012 | - | - | - | 0.270±0.042 | 0.030±0.009 | 0.080±0.014 |
|  | *d*_S_ | 0.050±0.034 | 0.011 | 0.025±0.012 | - | - | - | 0.128±0.039 | 0.041±0.013 | 0.060±0.015 |
|  | *P* | 0.026 | 0.540 | 0.371 | - | - | - | 0.044 | 0.465 | 0.328 |

a Only XXL was polymorphic at DQB2 and thus available for the calculation of evolutionary divergence; the other five populations were monomorphic at this locus.
