# Supplementary material for: Patterns of Adaptive and Neutral Diversity Identify the Xiaoxiangling Mountains as a Refuge for the Giant Panda
Source: PLoS One. 2013 Jul 19;8(7):e70229. doi: 10.1371/journal.pone.0070229 (PMC3716684; doi:10.1371/journal.pone.0070229)
Supplement: Table S7 — The likelihood ratio test of positive selection for the giant panda MHC genes. (DOC) [file pone.0070229.s008.doc]

Table S7 The likelihood ratio test of positive selection for the giant panda MHC genes.

|  | Models compared | Test statics | *P* |
| --- | --- | --- | --- |
| DQA1 | M1a vs M2a | 46.485 | <0.001 |
|  | M7 vs M8 | 46.502 | <0.001 |
| DQA2 | M1a vs M2a | 36.210 | <0.001 |
|  | M7 vs M8 | 36.212 | <0.001 |
| DRA | M1a vs M2a | 0.000 | 0.995 |
|  | M7 vs M8 | 0.000 | 0.995 |
| DQB1 | M1a vs M2a | 42.962 | <0.001 |
|  | M7 vs M8 | 103.674 | <0.001 |
| DQB2 | M1a vs M2a | 9.495 | <0.01 |
|  | M7 vs M8 | 9.797 | <0.01 |
| DRB3 | M1a vs M2a | 7.929 | <0.05 |
|  | M7 vs M8 | 8.316 | <0.05 |
